# Dietary yucca extract and *Clostridium butyricum* promote growth performance of weaned rabbits by improving nutrient digestibility, intestinal development, and microbial composition

**DOI:** 10.3389/fvets.2023.1088219

**Published:** 2023-02-13

**Authors:** Yuyan Wang, Yan Zhang, Hongjie Ren, Zubo Fan, Xu Yang, Cong Zhang, Yibao Jiang

**Affiliations:** ^1^College of Animal Science and Technology, Henan Agricultural University, Zhengzhou, Henan, China; ^2^College of Veterinary Medicine, Henan Agricultural University, Zhengzhou, Henan, China

**Keywords:** yucca extract, *Clostridium butyricum*, growth performance, meat quality, rabbit

## Abstract

*Yucca* has abundant amounts of polyphenolics, steroidal saponins, and resveratrol and its extract can be used as a feed additive in the animal husbandry, which might contribute to the improvement in the growth and productivity in rabbit production. Hence, the current study aimed to examine the effects of yucca extract alone and in combination with *Clostridium butyricum* (*C. butyricum*) on growth performance, nutrient digestibility, muscle quality, and intestinal development of weaned rabbits. A total of 400 40-day-old male rabbits were randomly divided into 4 treatment groups for 40 days: (1) basal diet group, (2) basal diet contained 300 mg/kg of yucca extract, (3) basal diet supplemented with 0.4 × 10^10^ colony-forming units (CFU)/kg of *C. butyricum*, and (4) the blend of 0.4 × 10^10^/kg CFU of *C. butyricum* and 300 mg/kg of yucca extract. The supplementation of yucca extract or *C. butyricum* increased body weight (BW) of rabbits depending on the age, the combined addition of yucca extract and *C. butyricum* significantly increased BW, weight gain, and feed intake, companying with increased the digestibility of crud protein, fiber, phosphorous, and calcium as compared to control diet (*P* < 0.05). Furthermore, yucca extract and *C. butyricum* treatment alone and in combination notably increased the villus high and the ratio of villus high to crypt depth of rabbits (*P* < 0.05). The combined supplementation of yucca extract and *C. butyricum* altered the intestinal microbiota of rabbits, as demonstrated by increased the abundance of beneficial bacteria *Ruminococcaceae* and decreased the proportion of pathogenic bacteria such as *Pseudomonadaceae* and *S24-7*. In addition, the rabbits fed the diet with yucca extract and the blend of yucca extract and *C. butyricum* had significantly increased pH_45min_, decreased pressing loss, drip loss, and shears force when compared with rabbits received control diet (*P* < 0.05). Diet with *C. butyricum* or its mixture with yucca extract increased the fat content of meat, while the combined addition of yucca extract and *C. butyricum* declined the content of fiber in meat (*P* < 0.05). Collectively, the combined use of yucca extract and *C. butyricum* showed better results on growth performance and meat quality, which might be closely associated with the improved intestinal development and cecal microflora of the rabbits.

## 1. Introduction

The transformation of weaned rabbits from breast milk to solid feed will cause many physiological and environmental stresses, which could lead to the prevalence and spread of enteric pathogens such as *Escherichia coli* and *coccidia*, and finally bring huge economic losses to animal husbandry due to negatively affect the growth performance and feed efficiency, as well as impair the animal welfare of rabbits. Considering the banned use of antibiotic growth promoters, mainly because of the emergence of bacteria resistant to multiple types of antibiotics, the development of antibiotic substitute and alternative feed additives to promote intestinal health, protect the stability of gastrointestinal microorganisms, and improve growth performance is urgent to the producers.

Emerging evidence have showed that plants and their extracts exert positive roles in animal growth, immunity, and maintaining animal health (1). The yucca extract contains saponins, polysaccharides, polyphenols, and other active substances (2), and is generally recognized as a safe feed additive. Studies have confirmed that yuca extract can enhance antioxidant function and immunity, maintain intestinal health, and improve animal growth performance in laying hens (3) and broilers (4). In addition, a diet with yucca extract can improve feed nutrient utilization, gut microflora, and intestinal barrier function of weaned piglets (5, 6). Dietary yucca extract was also found to promote female rabbit growth and fecundity through affecting the release of hormones and reducing ovarian resistance to benzene (7, 8). Of note, the performance of livestock is closely related to gut microbial load, the intestinal barrier, and the activity of the immune system, which could be regulated by probiotics (9). *Clostridium butyricum* (*C. butyricum*), a obligate anaerobic gram-positive probiotics that produces butyric acid (10), is considered to be one of the beneficial bacteria that widely colonized in animal intestines, and is proved to promote growth, strength immune system, and regulate intestinal microbial composition in weaned piglets (11), goats (12), and Peking ducks (13). These above evidence imply supplementation with yucca extract and/or *C. butyricum* probably improve the growth performance and health of rabbits.

Although single probiotic bacteria or plant extract have been widely applied in animal production, the application of combinational yucca extract and *C. butyricum* in the rabbit industry is rarely reported. The combined supplementation of probiotics and plant extract could be superior to them individual utilization as different species of probiotics or extract may promote animal health and production performance through different effects on gut microbial composition and cooperative action between different bacterial species and botanical extracts (14). However, some antagonism roles might be possible regarding the use of probiotics and plant extracts. In this context, diets with yucca extract alone or supplementation of clostridium butyric acid, are being practiced in the pigs (6, 11), poultry (3, 4), and ruminant animal research (12). The aim of this study, therefore, was to evaluate whether yucca extract and *C. butyricum* addition in rabbit feed improves growth, nutrient digestibility, meat quality, and intestinal microflora, which may provide some guidelines for the development of antibiotics alternative feed additives in rabbit production.

## 2. Materials and methods

### 2.1. Ethical statement

All experimental protocols were approved by the Animal Care and Use Committee of Henan Agricultural University and the animals were maintained in accordance with office guidelines for the care and use of laboratory animals (approval number: HN20210806).

### 2.2. Yucca extract and bacterial strain

Yucca schidigera extract with an active ingredient content of 60% was purchased from Xi'an Lutian Biotechnology Co., Ltd. (Xi'an, China). The *C. butyricum* was provided by Hubei Greensnow Biological Biotechnology Co., Ltd. (Wuhan, China) and the bacterial concentration reached 1 × 10^10^ colony-forming units (CFU)/g.

### 2.3. Animals, experimental design, and diets

To rule out the effects of gender, the just male rabbits from New Zealand White line (Henan, Jiyuan, China) were used in the present study. The animals were individually housed in metal cages (0.4 × 0.6 × 0.45 m) in a climate-controlled facility. The temperature in the room was 15–20°C with relative humidity was 55–65% based on normal management practices. The light schedule was 16-h light and 8-h dark throughout the experiment. The rabbits (40 days of age) with mean 1.05 ± 0.02 kg body weight (BW) were randomly into 4 treatment groups with 5 replicates of 20 rabbit each group, i.e., (1) basal diet group (Ctrl), which was formulated according to NRC (1977) rabbit feeding standard and shown in Table 1, (2) the basal diet supplemented with 400 mg *C. butyricum* per kg diet (0.4 × 10^10^) CFU/kg diet of *C. butyricum*, (3) the basal diet with 300 mg yucca schidigera extract per kg diet (YSE), and (4) the basal diet supplemented with both 400 mg *C. butyricum* and 300 mg yucca schidigera extract per kg diet (*C. butyricum* + YSE). The dosage of additives in this study were based on previous studies (15, 16). The rabbits were fed at 07:00 and 18:00 every day to ensure free access to drinking water and feed from 40 to 80 days.

**Table 1:** Composition and nutrient analysis of experimental diet (as-fed basis).

**Ingredients, %**		**Calculated analysis nutrient levels, %**	
Corn	17.0	Digestible energy (MJ/kg)	10.42
Bran	14.0	Crud protein	14.54
Soybean meal	16.0	Crud fiber	16.85
Alfalfa hay	4.0	Neutral detergent fiber	47.04
Peanut seedling	22.0	Acid detergent fiber	24.78
Peanut shells	9.0	Ether extract	5
Corn germ meal	15.0	Ash	9.81
Sodium chloride	0.5	Calcium	1.24
Calcium hydrogen phosphate	0.40	Total phosphorus	0.64
Stone powder	1.10		
Premix^a^	1		
Total	100		

a Premix is provided per kilogram of diet: 6,800 IU vitamin A; 1,200 IU vitamin D_3_; 9 mg vitamin E; 1.4 mg vitamin K; 0.5 mg vitamin B1; 1.75 mg vitamin B_2_; 18 mg niacin; 0.45 mg folic acid; 2.5 mg pantothenic acid; 0.09 mg biotin; 70 mg iron; 20 mg copper; 70 mg zinc; 10 mg manganese; 0.15 mg cobalt; 0.2 mg iodine; 0.25 mg selenium.

### 2.4. Growth performance

During the experiments, the feed intake and BW was recorded every 10 days after 8-h feed withdrawal. The average daily gain (ADG), average daily feed intake (ADFI), and feed to gain ratio (F:G) were calculated by recording the feed intake of rabbits in each pen. In addition, the number of rabbits with diarrhea in each replicate was recorded to calculate the diarrhea rate as following:


$$\text{Diarrhea rate = }\frac{\text{Nubmer of rabbits with diarrhea duing the trial}}{\text{Nubmer of test rabbits }\times \text{ Number of text days}}\;\times \;100$$

### 2.5. Determination of nutrient apparent digestibility

Five days before the end of the experiment, 3 rabbits from each replicate were randomly selected for digestion test. After a 3-day adaptation period, excreta from each cage were collected daily for the next 72 h. After each collection of feces, 10% hydrochloric acid was added to excreta nitrogen and stored at −20°C. After dry at 60°C for 72 h and ground to a size that could pass through a 1-mm screen, the feed and fecal samples were analyzed for dry matter (DM), ether extract (EE), crude protein (CP), neutral detergent fiber (NDF), acid detergent fiber (ADF), crude ash, calcium (Ca), and phosphorus (P), and then their apparent digestibility were determined as previous description (17).

### 2.6. Sample collection

On days 80, five similar BW rabbits from each treatment group were selected and sampled. After sacrification, the *longissimus thoracis* (LT) muscle from the left side of each carcass was used for the measurement of meat characteristics and composition. The middle segments of duodenum, jejunum, and ileum were stored in 4% paraformaldehyde for morphological analysis. Digesta samples of cecum were collected and stored in liquid N_2_ for 16S rDNA sequencing.

### 2.7. Muscle quality and nutrient composition

The meat characteristics included pH, water holding capacity (WHC) expressed as pressing loss (%), drip loss (%) and cooking loss rate (%), and tenderness, as well as the nutrient composition including CP, ether extract, and crude ash were analyzed based on the standard methods (18).

The pH_45min_ and pH_24h_ values were measured twice on LT at 45 min and 24 h postmortem, respectively, using a TESTO 205 pH acidity tester (Mettler-Toledo International Inc., USA) equipped with an insertion glass electrode. The pH meter was calibrated before measurements using standard phosphate buffers (pH = 4.01 and 7.00) and adjusted to the actual temperature of sample measurement following the instrumental user's manual.

The filter-paper press method was used to measure pressing loss. Cored LT samples, 2.523 cm in diameter and 1.0 cm in thickness, were collected and weighed (W1). Subsequently, the meat sample was placed on the pressure gauge platform and pressed to 35 kg (the range of the pressure gauge is about 138 kg) for 5 min. Samples were reweighed (W2) and pressing loss (%) was calculated according to the following equation: (W1–W2)/W1 × 100%.

Two pieces of about 5 g dorsal muscle were cut into 5 mm × 5 cm strips and weight (W3). Then the meat samples were placed in a water drop loss measuring tube avoiding sticking to the wall. After 24 h of the refrigerator at 4°C, these samples were removed, dried the surface moisture of muscle with filter paper, and weighed (W4), and the water drop loss (%) was calculated according to the following equation: (W3–W4)/W3 × 100%.

About 100 g of LT was subsampled by cutting 5 × 3 × 2 cm cubes devoid of fat and connective tissue and weighted (W5). Each cube was cooked in a water bath at 80°C until an internal temperature of 70°C was reached. Subsequently, the cooked samples were then cooled at 4°C for 2 h and reweighed (W6). The cooking loss (%) was calculated according to the following equation: (W5–W6)/W5 × 100%.

Tenderness was measured through the shears to force values and expressed in Newton (N) (19). Meat sample was putted into a constant temperature water bath at 80°C until the core temperature of the muscle reached 70°C, subsequently cooled at 4°C for overnight. About 6 to 8 1.27-cm-dia cylindrical cores parallel to the muscle fiber orientation were removed from each meat. The peak shear force measurement was obtained for 3–5 core each sample using a Warner-Bratzler meat shear machine (C-LM3B, Tenovo, Beijing, China) and the arithmetic mean was calculated for each meat sample.

### 2.8. Morphological analysis of small intestine

The fixed segments of duodenum, jejunum, and ileum were dehydrated, embedded, sliced into 5-μm transects, and stained with hematoxylin and eosin (H&E), and subsequently villus height (VH) and crypt depth (CD) of at least ten well-oriented villi, were measured and the ratio of villus height to crypt depth (V/C) was calculated. The histomorphometry data were taken using a microscope (Nikon Eclipse TS100; Nikon Corporation) and an image analyzer (Media Cybernetics Image Pro-Plus) at a magnification of 400×.

### 2.9. Gut microbiome analysis

The caecal content was mixed with lysis buffer which was composed of 40 mM ethylene diamine tetraacetic acid, 50 mM Tris pH 8.3, and 0.75 M sucrose, and then submitted to smooth shaking for 30 min. 200 μl of supernatant were used for the DNA extraction with the QIAamp DNA Stool kit (QIAGEN, Hilden, Germany). The quantity and quality of DNA was detected by using the Nanodrop ND-2000 spectrophotometer (Thermo Fisher Scientific, USA) and 1% agarose gel electrophoresis, respectively. The fusion primers 341F (5′-CCTACGGGNGGCWGCAG-3′) and 806R (5′-GGACTACHVGGGTATCTAAT-3′) were used to amplify the V3–V4 hypervariable region of the 16S rRNA gene using 200 ng DNA based on the 2-step PCR protocol. Sequencing library was prepared, and high-throughput sequencing was performed using the Illumina platform (Illumina, San Diego, US). Initial screening was conducted for the original off-machine data of high-throughput sequencing according to the sequence quality, and the problem samples were retested. Then the primer fragments of the sequence were removed, and the sequences of unmatched primers were discarded, and the steps of quality control, denoising, splicing and chimerism removal were carried out according to DADA2 analysis process in QIIME software (20). The alpha diversity was evaluated by calculating Chao1 estimator, Simpson, and Shannon diversity index. Beta-diversity was estimated by calculating the distance of dietary treatments to Ctrl group based on Bray-Curtis dissimilarities. Differentially enriched Kyoto Encyclopedia of Genes and Genomes (KEGG) functional pathways were also calculated.

### 2.10. Statistical analysis

In this study, the statistical power of 0.75 (75%) was obtained when the minimally detectable effect size was 1.0 and the significance level was 0.05. The data obtained were analyzed by the Shapiro-Wilk and Levene's test to assess normal distribution and homogeneity of variances (SPSS 26.0). One-way analysis of variance (ANOVA) by Duncan test for multiple comparisons and Kruskal-Wallis test followed by Dunn's multiple comparisons were performed for normal distribution and non-normal distribution, respectively. Values are given as mean ± standard deviation. *P* < 0.05 was considered statistically significant.

For the determination of the growth curves of BW of rabbits, three non-linear regression model (von Bertalanffy, logistic, and Gompertz) were assessed based on the coefficient of determination (*R*^2^) using the non-linear models (PROC NLIN) of SAS by the Gauss-Newton algorithm (data not shown). The logistic model was finally selected as the optimized model for BW as the equation: BW = *a*/(1 – *b* × EXP(–k^*^day)), *a* is the asymptotic value of modeled trait, *b* is a constant of integration without biological interpretation, *k* is the maturity rate. The age of maximal growth rate is on lnb/*k* day.

## 3. Results

### 3.1. Growth performance

**Figure 1:**
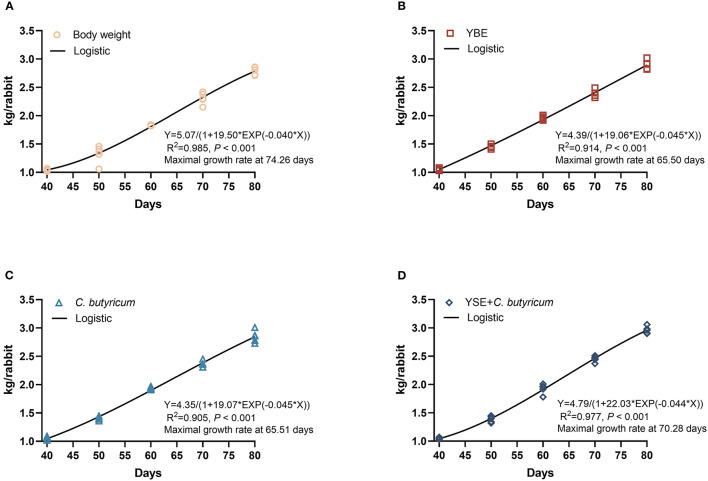
**(A)** Body weight of Ctrl. **(B)** Body weight of YSE. **(C)** Body weight of *C. butyricum*. **(D)** Body weight of YSE+*C. butyricum*. Body weight of rabbits at different days in response to dietary treatments with and logistic model: BW = *a*/(1 – *b* × EXP(–k*day)), in which a is the asymptotic value of modeled trait, b is a constant of integration without biological interpretation, *k* is the maturity rate. The age of maximal growth rate is on lnb/*k* day.

The effects on the growth performance are showed in Table 2, when compared with Ctrl group, dietary YSE inclusion increased the BW of rabbits on days 50, and *C. butyricum* or YSE treatment significantly increased the BW of rabbits on 60 days (both *P* < 0.05). Of note, diet with *C. butyricum* and YSE induced a remarkably increase in BW than the Ctrl diet on days 70 and 80 (*P* < 0.05), which was consistent with the ADG results (*P* < 0.05). Furthermore, based on these growth curve showed in Figures 1A–D, the ages of maximal growth rate of YSE, *C. butyricum*, and YSE + *C. butyricum* were earlier than those in Ctrl group, i.e., the age of maximal growth rate was 74.36, 65.50, 65.51, and 70.28 days in Ctrl, YSE, *C. butyricum*, and YSE + *C. butyricum* groups, respectively. Regarding feed consumption, the rabbits fed *C. butyricum* or YSE diet presented a significant increase in ADFI during 40–50 and 51–60 days as compared to those received Ctrl diet. The combined supplementation of *C. butyricum* and YSE significantly increased the ADFI during the whole study period (40–80 days). Except the decreased ration of feed to gain in YSE and *C. butyricum* + YSE group, the dietary administration not significantly changed the F/G when compared to Ctrl group. In addition, dietary supplemented with YSE, *C. butyricum*, or their blend could decrease the diarrhea rate to varying degrees when compared to Ctrl diet.

**Table 2:** Effects of yucca extract (YSE) and *C. butyricum* on growth performance and diarrhea rate of rabbits.

**Item**	**Ctrl**	**YSE**	***C. butyricum***	***C. butyricum* + YSE**	***P-*value**
**Body weight, kg/rabbit**					
40 days	1.05 ± 0.02	1.05 ± 0.02	1.05 ± 0.03	1.05 ± 0.01	0.992
50 days	1.32 ± 0.16^b^	1.45 ± 0.03^a^	1.41 ± 0.03^ab^	1.39 ± 0.06^ab^	0.176
60 days	1.83 ± 0.01^b^	1.96 ± 0.03^a^	1.93 ± 0.02^a^	1.93 ± 0.09^a^	0.003
70 days	2.31 ± 0.10^b^	2.39 ± 0.06^ab^	2.36 ± 0.05^ab^	2.45 ± 0.05^a^	0.038
80 days	2.79 ± 0.07^b^	2.90 ± 0.08^ab^	2.85 ± 0.11^ab^	2.96 ± 0.07^a^	0.031
**Average daily gain, g/rabbit**					
40–50 days	31.60 ± 7.40^b^	39.14 ± 3.25^a^	35.98 ± 4.06^ab^	34.53 ± 6.00^ab^	0.214
51–60 days	39.28 ± 7.88^b^	46.15 ± 6.11^ab^	47.76 ± 4.35^ab^	50.43 ± 5.82^a^	0.063
61–70 days	33.91 ± 5.39^b^	36.15 ± 1.45^ab^	39.27 ± 7.71^ab^	43.07 ± 6.16^a^	0.1
71–80 days	44.49 ± 3.82	44.90 ± 6.57	41.86 ± 4.97	46.15 ± 3.10	0.562
40–80 days	37.32 ± 2.86^b^	41.58 ± 2.40^a^	41.21 ± 3.31^ab^	43.54 ± 3.05^a^	0.027
**Average daily feed intake, g/rabbit**					
40–50 days	110.71 ± 7.09^b^	116.91 ± 4.82^ab^	122.29 ± 5.35^a^	117.61 ± 3.52^ab^	0.028
51–60 days	177.18 ± 4.24^b^	176.99 ± 8.78^b^	196.19 ± 6.90^a^	186.09 ± 7.00^b^	0.001
61–70 days	187.59 ± 26.94	207.40 ± 10.35	214.37 ± 27.57	219.32 ± 21.25	0.17
71–80 days	207.50 ± 29.21	199.58 ± 26.65	200.98 ± 10.25	219.02 ± 10.72	0.471
40–80 days	170.75 ± 10.26^b^	175.22 ± 10.73^ab^	183.46 ± 8.46^ab^	185.51 ± 6.16^a^	0.066
**Fend intake/Gain, g/g**					
40–50 days	3.67 ± 0.92	3.00 ± 0.15	3.42 ± 0.27	3.48 ± 0.56	0.307
51–60 days	4.62 ± 0.70^a^	3.88 ± 0.43^b^	4.13 ± 0.34^ab^	3.72 ± 0.35^b^	0.045
61–70 days	5.44 ± 0.68	5.74 ± 0.12	5.52 ± 0.42	5.12 ± 0.34	0.214
71–80 days	5.59 ± 0.59	5.47 ± 1.37	5.84 ± 0.84	5.70 ± 0.17	0.918
40–80 days	4.83 ± 0.41	4.52 ± 0.28	4.73 ± 0.27	4.51 ± 0.19	0.267
**Diarrhea rate, %**					
40–80 days	2.73 ± 1.31^a^	1.25 ± 0.33^b^	1.70 ± 1.28^ab^	1.55 ± 0.71^ab^	0.146

Means within a row with different superscripts are significantly different (*n* = 100; *P* < 0.05).

### 3.2. Nutrient apparent digestibility

As illustrated in Table 3, diets with YSE, *C. butyricum*, or their compound notably increased the digestibility of CP, ADF, and Ca as compared to Ctrl diet (both *P* < 0.05). Supplementation of the blend of YSE and *C. butyricum* also significantly elevated the digestibility of NDF and P (*P* < 0.05). There was no significant difference in the digestibility of EE among all groups (Table 3).

**Table 3:** Effects of yucca extract (YSE) and *C. butyricum* on nutrient apparent digestibility of rabbits.

**Item, %**	**Ctrl**	**YSE**	***C. butyricum***	***C. butyricum* + YSE**	***P-*value**
Ether extract	76.78 ± 6.21	79.32 ± 8.39	77.82 ± 9.66	81.19 ± 12.25	0.943
Crude protein	68.12 ± 1.50^c^	69.91 ± 0.78^b^	71.86 ± 0.19^a^	71.13 ± 0.41^ab^	0.004
Neutral detergent fiber	32.98 ± 1.40^b^	34.17 ± 1.33^ab^	34.81 ± 0.52^ab^	35.32 ± 0.35^a^	0.097
Acid detergent fiber	23.19 ± 1.20^b^	27.39 ± 1.17^a^	28.16 ± 2.23^a^	27.22 ± 1.30^a^	0.016
Ash	42.59 ± 0.35^ab^	41.75 ± 1.27^b^	42.91 ± 3.42^ab^	45.72 ± 0.81^a^	0.132
Calcium	58.00 ± 5.41^b^	64.56 ± 2.58^a^	67.69 ± 2.62^a^	65.85 ± 2.11^a^	0.039
Phosphorus	27.42 ± 1.02^b^	31.13 ± 2.10^b^	33.13 ± 2.46^ab^	37.47 ± 4.86^a^	0.019

Means within a row with different superscripts are significantly different (*n* = 5; *P* < 0.05).

### 3.3. Small intestinal morphology

**Figure 2:**
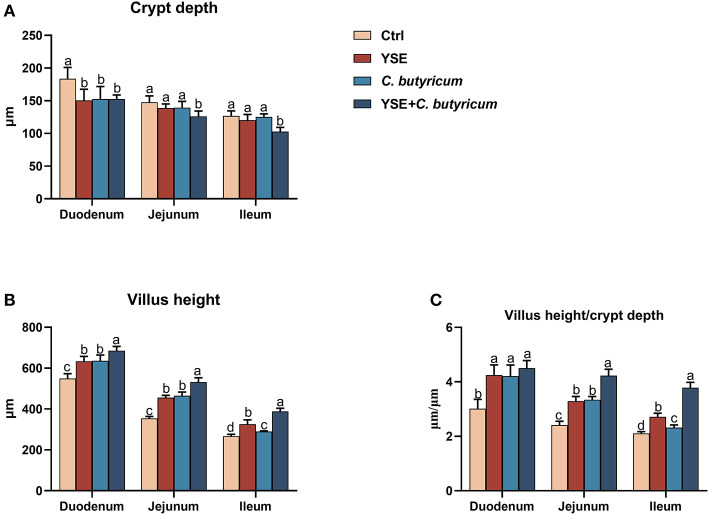
Dietary yucca extract (YSE) and *C. butyricum* promote the development of small intestine in rabbits. **(A)** Crypt depth and **(B)** villus height of duodenum, jejunum, and ileum were measured, and **(C)** the ratio of villi height to crypt depth were calculated based on hematoxylin/eosin (H&E) staining. Scale bar = 100 μm. Values are means and standard deviation (SD) represented by vertical bars. ^a, b^Mean values with different letters are significantly different (*n* = 5; *P* < 0.05).

The diet with YSE or *C. butyricum* reduced duodenal CD, while it did not apparent change the CD of jejunum and ileum (Figure 2A). Dietary supplementation of both YSE and *C.butyricum* could decrease the intestinal CD when compared to Ctrl diet. Rabbits fed *C. butyricum* or YSE had a higher VH and VH/CD of duodenum, jejunum, and ileum than the those fed the Ctrl diet. In particular, the combined supplementation of YSE and *C. butyricum* contributed higher villus and VH/CD than single YSE or *C. butyricum* group (Figures 2B, C).

### 3.4. Muscle quality and nutrient composition

The effects of YSE and *C. butyricum* on muscle quality and nutritional composition of LT are shown in Table 4. The rabbits in the YSE- and YSE + *C. butyricum*-supplemented groups had significantly increased pH_45min_ compared with rabbits in the Ctrl group (*P* < 0.05). The dietary YSE and *C. butyricum* supplementation improved the WHC of LT, evidenced by decreased pressing loss and drip loss in YSE, *C. butyricum*, and YSE + *C. butyricum* as compared with that in the Ctrl group. In addition, the meat shears force of *C. butyricum*, and YSE + *C. butyricum* groups was significantly lower than that of the Ctrl group (*P* = 0.033). In terms of nutrient composition of meat, diet with *C. butyricum* or the blend of YSE and *C. butyricum* increased the EE content of LT, whereas the dietary treatments did not change the content of moisture, CP, and ash in meat when compared with the Ctrl diet (*P* < 0.05, Table 4).

**Table 4:** Effects of yucca extract (YSE) and *C. butyricum* on meat quality of rabbits.

**Item**	**Ctrl**	**YSE**	***C. butyricum***	***C. butyricum* + YSE**	***P-*value**
**Meat quality traits**					
pH_45min_	6.05 ± 0.13^b^	6.67 ± 0.19^a^	6.28 ± 0.22^b^	6.66 ± 0.19^a^	0
pH_24h_	5.72 ± 0.15	5.71 ± 0.14	5.68 ± 0.17	5.74 ± 0.04	0.9
Pressing loss, %	29.87 ± 3.81^a^	20.89 ± 4.63^b^	24.37 ± 5.90^ab^	20.42 ± 4.51^b^	0.023
Drip loss, %	4.01 ± 0.38^a^	1.72 ± 0.12^c^	2.82 ± 0.24^b^	1.43 ± 0.54^c^	0
Cooking loss, %	27.21 ± 1.47	25.19 ± 4.41	26.56 ± 3.03	24.97 ± 4.13	0.697
Shear force value, N	41.54 ± 4.00^a^	37.78 ± 2.86^ab^	35.40 ± 4.06^b^	34.33 ± 3.72^b^	0.033
**Meat composition, %**					
Moisture	74.49 ± 0.86	75.19 ± 1.12	74.60 ± 0.98	74.76 ± 0.45	0.633
Ether extract	4.19 ± 1.10^b^	5.18 ± 0.82^b^	6.78 ± 0.86^a^	6.60 ± 0.69^a^	0.001
Crude protein	83.24 ± 1.85	84.05 ± 1.56	83.62 ± 1.21	84.08 ± 1.37	0.793
Ash	4.83 ± 0.25	5.02 ± 0.17	4.84 ± 0.30	4.68 ± 0.29	0.255

Means within a row with different superscripts are significantly different (n = 5; P < 0.05).

### 3.5. Microbiota structure in colonic contents

**Figure 3:**
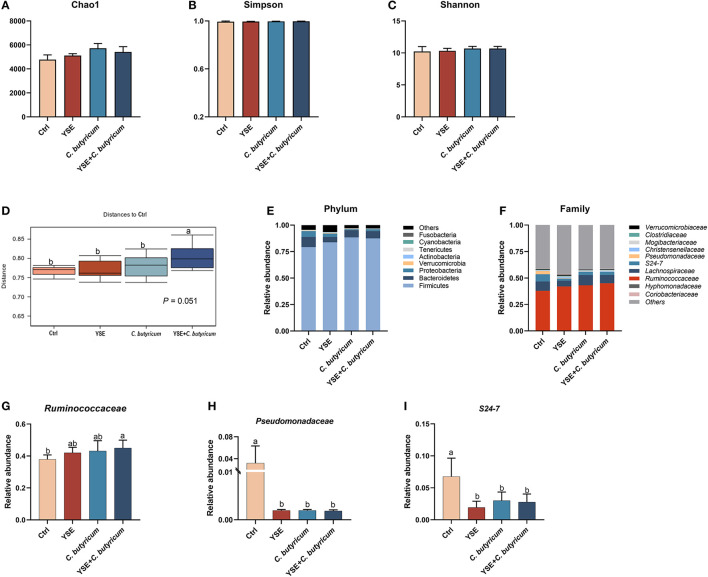
Dietary yucca extract (YSE) and *C. butyricum* on caecal microbiome of rabbits. **(A–C)** Chao1, Simpson, and Shannon indexes were used to assess alpha diversity, **(D)** the distance to Ctrl group of caecum microbiome diversity at species level based on Bray-Curtis dissimilarities; the relative abundances of bacterial communities at **(E)** phylum level and **(F)** family level, including **(G)** *Ruminococcaceae*, **(H)** *Pseudomonadaceae*, and **(I)** *S24-7*. Values are means and standard deviation (SD) represented by vertical bars. ^a, b^Mean values with different letters are significantly different (*n* = 5; *P* < 0.05).

**Figure 4:**
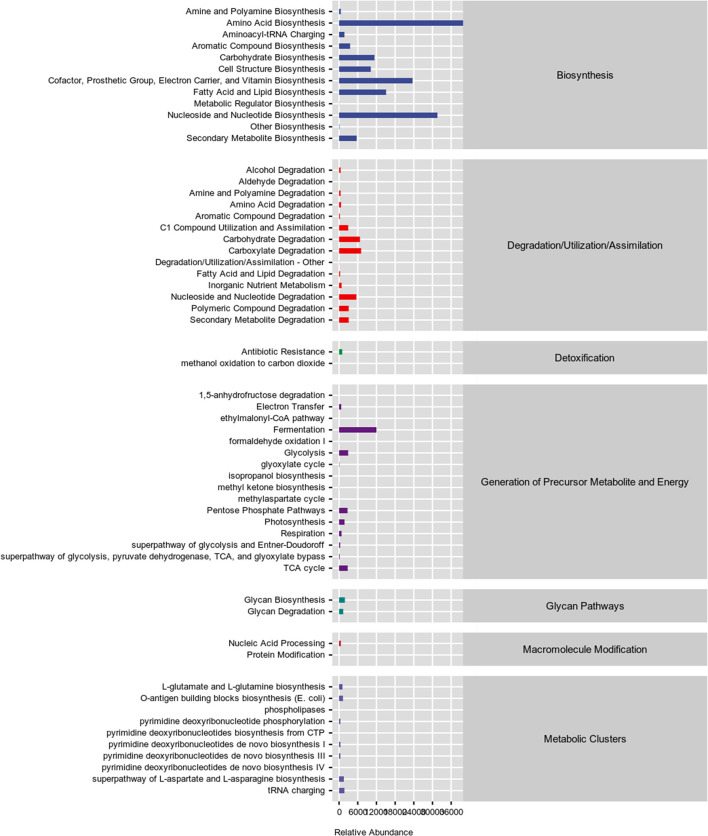
Functional predictions for the caecal microbiome based on Kyoto Encyclopedia of Genes and Genomes (KEGG) analysis.

The microbiota in the colonic content is presented in Figure 3. The dietary interferes did not change the alpha diversity, showed by similar Chao1, Simpson, and Shannon indexes (Figures 3A–C; *P* > 0.05). The administration of YSE combined *C. butyricum* induced an apparent difference in microbiota composition of the colonic contents in rabbits (Figure 3D), which mainly comprised Firmicutes, Bacteroidetes, Proteobacteria, and Tenericutes at the phylum level (Figure 3E). At the family level, *Ruminococcaceae* was the dominant species in the cecal flora of rabbits, with 37.94% abundance in the Ctrl group, 43.11% abundance in the *C. butyricum* group, 41.97% abundance in YSE group and 45.05% abundance in the combined treatment group (Figures 3F, G). Dietary supplementation with YSE and *C. butyricum* increased the abundance of beneficial bacteria *Ruminococcaceae* and decreased the proportion of pathogenic bacteria *Pseudomonadaceae* and *S24-7* (Figures 3G–I). In addition, as illustrated in Figure 4, the metabolic pathways were predicted using KEGG according to the known microbial genome data, and these results showed that dietary YSE or/and *C. butyricum* mainly affected biosynthesis including amino acid, nucleoside, and fatty acid, etc.

## 4. Discussion

The physiological and environmental stress of rabbits due to weaning usually occur during the initial postweaning period, which is frequently characterized by transient anorexia, gut microbiota dysbiosis, severe intestinal damage, infections, and diarrhea, compromising the disease resistance of juvenile rabbit (21). Reasonable nutritional regulation means are very important for the normal growth of weaned rabbits on this. In this study, dietary supplementation with the blend of yucca extracts and *C. butyricum* had a positive role in growth performance, digestive ability, and meat quality of rabbits, which might involve in the improved intestinal development and intestinal microbiota.

*Yucca* is rich in polyphenols, steroidal saponins and resveratrol, which was often used in food, cosmetics, pharmaceutical and animal feed as a solution or powder due to its properties of antioxidant, anti-inflammatory, antiviral, lowering cholesterol (22). The addition of yucca extract to feed can promote the growth performance of rabbits (7) and broilers (4, 23). Additionally, the butyrate produced by *C. butyricum* is essential for the proper functioning of gut. Treatment with *C. butyricum* could promote growth and regulate intestinal microbial composition in weaned piglets (11), goats (12), and ducks (13). Diet with *C. butyricum* was proved to improve intestinal morphology, gut microbiota, and growth performance of rabbits (24). Consistent with previous findings, the outcomes of performance indicated that the ages of maximal growth rate of YSE, *C. butyricum*, and YSE + *C. butyricum* were earlier than those in Ctrl group. Diet with *C. butyricum* or yucca extract increased the BW of rabbits depending on the age, while the combined supplementation of *C. butyricum* and yucca extract in feed significantly increased the BW and ADFI during 40–80 days in the present study, which suggests that dietary supplementation of yucca extract has synergetic effect with *C. butyricum* and enhanced the growth performance of rabbits.

Improving nutrient apparent digestibility might be a key reason to explain these positive roles of dietary combined supplementation yucca extract and *C. butyricum* in growth performance of rabbits, evidenced by the increased digestibility of CP, ADF, NDF, P, and Ca in YSE + *C. butyricum* group. Of note, the direct action of the combined supplementation yucca extract and *C. butyricum* on digestive physiology, regulation of the intestinal development, and remodeling gut microbiota might be closely associated with the improved digestibility of rabbits. It was reported that steroidal saponins in yucca extract could increase the activity of digestive enzymes and have a positive promoting effect on digestive tract (25). Polyphenols existing yucca extract was found to increase the attention of nutrition in the digestive tract due to their multiple bioactive properties (26). Therefore, dietary yucca extract addition increased the digestibility of fat and DM of sows during late gestation and lactation (27), as well as improved feed conversion, protein efficiency, and energy efficiency of broilers (28). In addition, *C. butyricum* is also pointed to increase the activity of digestive enzymes, and ultimately improving the digestion and absorption of nutrients (29), which might contribute to the feed consumption of rabbits during 40–60 days.

Promotion of intestinal development might be another likely explanation for the elevation in nutrient apparent digestibility in the current study. Although exerting immunologic function, intestinal tract mainly involves in digestion and absorption of nutrition. The length and thickness of villi directly affect the absorption area of the intestinal tract, and then affect the absorption and utilization of nutrients by animals (30). The longer intestinal villi and the shallower crypt depth indicate the stronger absorption capacity of nutrients. It was noticed that dietary yucca extract increased the villus height and the ratio of villus height to crypt depth in ileum of weaned piglets (6). Administration with *C. butyricum* as a probiotic in the diet could increase the ratio of villus height to crypt depth of geese (31). Analogously, in this study, the supplementation alone or combined yucca extract and *C. butyricum* notably increased the villus height and the ratio of villus height to crypt depth. It probably increases the ability of intestinal digestion and absorption of nutrients and improved the performance of rabbits.

Intestinal microbiota plays an important role in the development of the immune system and the maintenance of the intestinal barrier. Dysbiosis of intestinal flora can lead to the destruction of the intestinal barrier and increase the susceptibility to pathogenic microorganism (32). Dietary yucca extract and *C. butyricum* induced significant changes in microbial composition as evidenced by a large distance between YSE + *C. butyricum* and Ctrl groups in this study. At the family level, *S24-7, Ruminococcaceae*, and *Lachnospiraceae* were the most abundant in rabbits. S24-7 is negatively correlated with BW and had long-term adverse effects on growth (33), and plays important roles in amino acid metabolism and intestinal mucosal immunity (34). The decreased relative abundance of *S24-7* implied the improved role of the addition of yucca extract and *C. butyricum* to rabbit feed. The mechanism of antiprotozoal effects of yucca extract is the formation of irreversible complex between saponins and cholesterol (25). In addition, *C. butyricum* also decreased the abundance of pathogenic bacteria and increased the abundance of beneficial bacteria to regulate the composition of intestinal flora (11). Previous data showed that *C. butyricum* could promote the growth and reproduction of cellulolytic bacteria and fungi in gut, improve cellulase activity and thus increase the digestibility of cellulose and ADF in goats (35). In this study, dietary supplementation with YSE and *C. butyricum* increased the abundance of beneficial bacteria *Ruminococcaceae* and decreased the proportion of pathogenic bacteria *Pseudomonadaceae* in gut microbiota might be closely related to nutrient digestion in rabbits, especially NDF and ADF. Further studies are needed to confirm the possibility.

Meat quality traits such as pH, color, WHC, and tenderness are critical to consumers' initial selection of rabbit meat as well as for final product satisfaction. As a key indicator of the glycolysis rate of muscle glycogen after slaughter, the pH of meat is gradually reduced as the slaughtering time goes on, and too low pH value could cause the meat to be rotten and soft. In this regard, increased pH_45min_ in YSE, *C. butyricum*, and YSE + *C. butyricum* diets indicated the positive role of yucca extract and *C. butyricum* in meat quality of rabbits. It is well-known that lower pH prompts muscle fiber contraction, causing more drip loss (36), thus the increased pH might explain why the enhancement in WHC of meat by the diet contained yucca extract and *C. butyricum* in the current study, evidenced by lower pressing loss and drip loss. Similarly, feeding the diet with yucca extract could reduce the water loss rate of broiler muscle (37). Dietary *C. butyricum* treatment was also found to improve the pH_45min_ of meat in 28-day-old Huanjiang Mini-Pigs (38), and reduce drip loss of pectoral muscle in Peking ducks (13). In addition, shear force is negatively correlated with the tenderness of the muscle, which is affected by multiple factors including pH, WHC, postmortem proteolysis, meat composition such as fat and fiber (39, 40). In this study, diet with yucca extract and *C. butyricum* increased the tenderness of meat in rabbits, which could attribute to higher fat proportion. Of note, the failure of yucca extract to increase muscle fat may be due to the inhibitory effect of saponins on pancreatic lipase activity. Saponins can reduce the fat rate of meat by inhibiting pancreatic lipase activity and delaying muscle fat deposition (41). Taken together, the combined addition of yucca extract and *C. butyricum* to feed could improve meat quality of TL in rabbits.

## 5. Conclusions

In summary, using yucca extract and *C. butyricum* as feed additives could increase nutrient digestibility and improve growth performance, which is linked to the alteration in digestive physiology, intestinal development, and gut microbiota. In addition, the combination of yucca extract and *C. butyricum* in feed has a synergistic effect on meat quality through increasing pH_45min_, WHC, and tenderness, as well as increasing fat proportion of meat.

## Data availability statement

The datasets presented in this study can be found in online repositories. The names of the repository/repositories and accession number(s) can be found below: https://www.ncbi.nlm.nih.gov/, PRJNA897093.

## Ethics statement

The animal study was reviewed and approved by Animal Care and Use Committee of Henan Agricultural University.

## Author contributions

YW collected data and wrote manuscripts under the guidance of YJ. YZ, HR, ZF, XY, and CZ helped to collect the literature. All authors contributed to the article and have approved the submitted version.
